# Screening, docking, and molecular dynamics analysis of Mitragyna speciosa (Korth.) compounds for targeting HER2 in breast cancer

**DOI:** 10.1016/j.crstbi.2025.100171

**Published:** 2025-06-20

**Authors:** Nabila Hadiah Akbar, Farendina Suarantika, Taufik Muhammad Fakih, Ariranur Haniffadli, Khoirunnisa Muslimawati, Aditya Maulana Perdana Putra

**Affiliations:** a Pharmacist Professional Education Study Program (PPSP), Faculty of Mathematics and Natural Sciences, Universitas Lambung Mangkurat, Jl. Brig Jend. Hasan Basri, Banjarmasin, South Kalimantan, 70123, Indonesia; b Pharmacist Professional Education Study Program (PPSP), Faculty of Mathematics and Natural Sciences, Universitas Islam Bandung, Jl. Batik Halus, Bandung, West Java, 40123, Indonesia; c Department of Pharmacy, Faculty of Mathematics and Natural Sciences, Universitas Islam Bandung, Jl. Ranggagading, Bandung, West Java, 40116, Indonesia; d Korean Medicine Convergence Science Major of KIOM School, University of Science and Technology (UST), Daejeon, 34113, Republic of Korea; e Herbal Medicine Resources Research Center, Korea Institute of Oriental Medicine (KIOM), 111 Geonjaero, Naju-si, Jollanam-do, 58245, Republic of Korea; f Department of Pharmacy, Faculty of Mathematics and Natural Sciences, Universitas Lambung Mangkurat, Jl. Brig Jend. Hasan Basri, Banjarmasin, South Kalimantan, 70123, Indonesia

**Keywords:** Breast cancer (BC), Human epidermal growth factor receptor 2 (HER2), Mitragyna speciosa (Korth.), Natural compounds screening, Molecular interactions approach

## Abstract

Breast cancer remains the most commonly diagnosed cancer among women worldwide, with approximately 2.3 million new cases reported in 2022. In the United States alone, an estimated 310,720 new cases of female breast cancer are expected in 2024. HER2-positive breast cancer, characterized by the overexpression of the human epidermal growth factor receptor 2 (HER2), accounts for about 20 % of all breast cancer cases. The development of anti-HER2 therapies has significantly improved survival rates for patients with HER2-positive breast cancer. In this study, we employed in silico methods to evaluate the potential of natural alkaloids, Mitragynine and 7-Hydroxymitragynine, as HER2 inhibitors. Molecular docking revealed binding energies of −7.56 kcal/mol and −8.77 kcal/mol, respectively, with key interactions involving residues such as Leu726, Val734, Ala751, Lys753, Thr798, and Asp863. Molecular dynamics simulations demonstrated the stability of all three complexes, including Mitragynine, 7-Hydroxymitragynine, and Native (SYR127063), over the simulation period. Mitragynine exhibited stronger interaction stability, supported by a higher hydrogen bond occupancy of 39.19 %, compared to 4.32 % for 7-Hydroxymitragynine, while Native (SYR127063) displayed the highest occupancy at 49.66 %. MM-PBSA analysis further validated these findings, with Native (SYR127063) exhibiting the most favorable total binding energy of −163.448 ± 17.288 kJ/mol, followed by Mitragynine at −112.33 ± 22.41 kJ/mol, and 7-Hydroxymitragynine at −103.56 ± 15.61 kJ/mol. ADMET, physicochemical properties, and drug-likeness evaluations indicated that all compounds satisfy Lipinski, Ghose, Veber, Egan, and Muegge rules, confirming their suitability as lead-like molecules. Based on these findings, Mitragynine and 7-Hydroxymitragynine are promising candidates for HER2-targeted breast cancer therapy, with further experimental validation recommended to confirm their clinical potential.

## Graphical abstract

**Figure:**
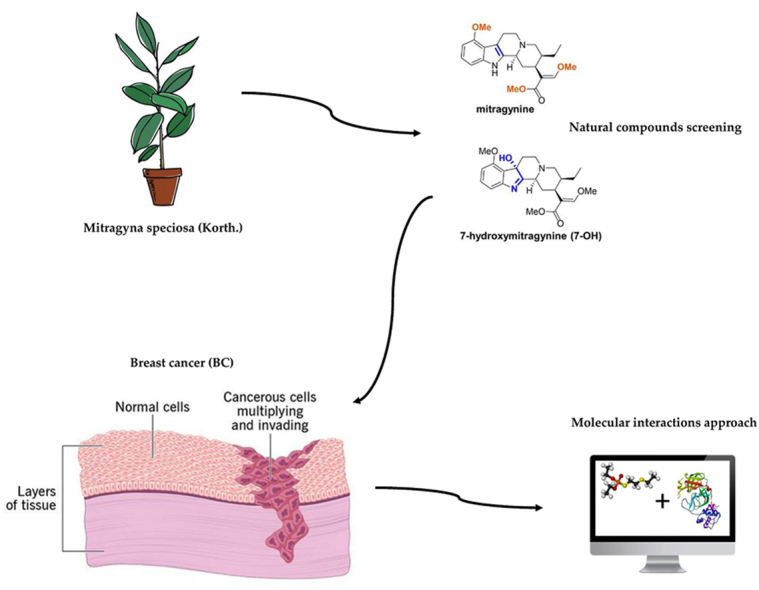

## 1 Introduction

Breast cancer (BC) remains the most frequently diagnosed cancer in women and is a leading cause of cancer-related deaths globally. According to recent statistics, approximately 2.3 million new cases of BC were reported worldwide in 2022, resulting in over 670,000 deaths, making it one of the most significant public health concerns (DeSantis et al., 2014). In the United States alone, projections for 2023 estimate 297,790 new invasive BC cases in women and 2800 cases in men (Parkin and Fernández, 2006; Miller et al., 2022). Risk factors such as advancing age, family history, genetic mutations, and lifestyle habits, including high-fat diets and excessive alcohol consumption, contribute to the growing incidence of BC. Early detection methods like breast self-examinations, mammography, and magnetic resonance imaging have greatly improved diagnosis and survival rates (Bonsu and Ncama, 2019; Rai, 2023). However, disparities in access to diagnostic tools and healthcare resources persist, particularly in low-income countries, leading to delays in treatment and worse prognoses. Despite advancements, the burden of BC continues to rise, necessitating innovative strategies for prevention, early detection, and therapeutic intervention (Kwan et al., 2023; Iacob et al., 2024). Addressing these challenges is critical for reducing BC mortality rates and improving the quality of life for affected individuals.

The human epidermal growth factor receptor 2 (HER2) is a well-established therapeutic target for breast cancer. HER2 overexpression, observed in approximately 15–20 % of BC cases, is strongly associated with aggressive tumor biology and poor clinical outcomes (Budi et al., 2022). This transmembrane receptor plays a critical role in tumor growth, proliferation, and metastasis by activating downstream signaling pathways. Current HER2-targeted therapies, such as trastuzumab and pertuzumab, have significantly improved patient survival rates. However, resistance to these therapies and associated adverse effects remain significant challenges in BC management (Iqbal and Iqbal, 2014; Nixon et al., 2018). The need for novel HER2-targeted inhibitors that overcome resistance and improve specificity has become a key focus of research. Advances in molecular biology and computational techniques have facilitated the identification of promising HER2 inhibitors (Siena et al., 2018; Erickson et al., 2020). These developments highlight the potential for innovative therapies to address unmet clinical needs in BC treatment. Understanding the structural and functional dynamics of HER2 is essential for developing new strategies to combat HER2-positive BC effectively.

Natural products have been a cornerstone of drug discovery, offering structurally diverse and biologically active scaffolds for therapeutic development. Among these, Mitragyna speciosa (Korth.), commonly known as kratom, has gained attention for its pharmacological properties (Ramanathan and McCurdy, 2020). Native to Southeast Asia, kratom has been traditionally used for its analgesic, stimulant, and anti-inflammatory effects. Recent studies have highlighted its potential anticancer properties, particularly through its bioactive alkaloids and terpenoids (Ramachandram et al., 2020; Firmansyah et al., 2021). Mitragynine, the primary alkaloid in kratom, has demonstrated activity against various cancer cell lines and may serve as a foundation for developing novel anticancer agents. Previous computational studies have also identified kratom-derived compounds as potential inhibitors of estrogen receptors and other cancer-related targets (Leksungnoen et al., 2022; Zhang et al., 2023). These findings suggest that Mitragyna speciosa could be a valuable source of natural compounds for HER2-targeted drug development. Leveraging its phytochemical diversity, this study explores the potential of kratom as a source of novel HER2 inhibitors.

While the direct inhibitory effects of Mitragyna speciosa alkaloids on HER2 have yet to be confirmed through in vitro assays, existing evidence supports their broad cytotoxic potential. Several preclinical studies have demonstrated that kratom extracts and mitragynine exhibit cytotoxicity against a range of cancer cell lines, including MCF-7 (breast cancer), SH-SY5Y (neuroblastoma), K562 (leukemia), HT-29 (colon cancer), A549 (lung cancer), HepG2 (liver cancer), and HeLa (cervical cancer), as well as MCL-5 (lymphoblastoid) and normal Vero cells (Priatna et al., 2024). However, it is important to note that these studies were not conducted in HER2-overexpressing models such as SK-BR-3 or BT-474. For example, MCF-7 cells are estrogen receptor-positive but HER2-negative (Lattrich et al., 2008). Consequently, while these findings establish the general anticancer potential of kratom-derived alkaloids, they do not directly validate HER2 as the molecular target. Nonetheless, kratom alkaloids such as mitragynine and 7-hydroxymitragynine are known to interact with diverse receptor systems including opioid and adrenergic receptors which supports the hypothesis that they may also engage receptor tyrosine kinases like HER2 (Hanapi et al., 2021). While further biological validation using HER2-overexpressing cell lines is required, these findings provide a reasonable foundation for computational evaluation of kratom-derived compounds as candidate HER2 inhibitors.

Computational approaches, such as virtual screening and molecular docking, have emerged as powerful tools in drug discovery. These methods allow the efficient identification of potential drug candidates by screening large libraries of natural compounds against specific therapeutic targets (Suenaga et al., 2012; Thamim et al., 2023). Docking-based screening, in particular, has proven effective in identifying promising HER2 inhibitors by predicting the binding interactions between ligands and the receptor. Molecular dynamics simulations further refine these predictions by assessing the stability and dynamics of ligand-receptor interactions. Recent studies have successfully applied these techniques to discover HER2 inhibitors with high binding affinities and favorable pharmacokinetic profiles. This study employs these computational methods to screen a library of natural compounds derived from Mitragyna speciosa against HER2. The aim is to identify potential lead compounds that could serve as a basis for developing more effective and specific HER2-targeted therapies. By integrating advanced computational techniques with the phytochemical richness of Mitragyna speciosa, this research seeks to contribute to the ongoing efforts in breast cancer drug discovery.

## 2 Materials and methods

### 2.1 Protein preparation

The crystal structure of the kinase domain of human HER2 was retrieved from the Protein Data Bank (PDB) using the PDB ID: 3PP0 (Aertgeerts et al., 2011). The structure file, in pdb format, was carefully inspected and prepared for the docking studies. Any missing side chain residues were identified and repaired using the Swiss-PDB Viewer tool (Guex et al., 2009). To ensure accuracy, the repaired structure underwent energy minimization to resolve any steric clashes or irregularities introduced during the preparation process. Non-essential molecules, such as water and co-crystallized ligands, were removed to avoid interference during docking. The processed structure was validated and saved in pdb format, ensuring compatibility with subsequent computational analyses. This optimized protein structure served as a robust model for evaluating interactions with the selected natural compounds.

### 2.2 Ligand selection

**Fig. 1:**
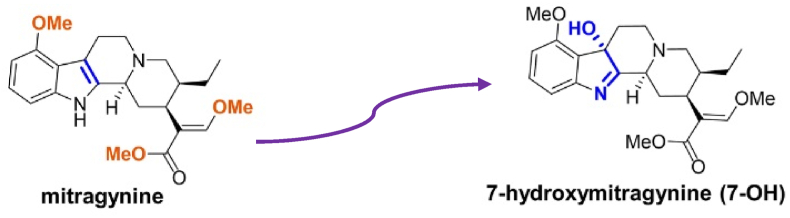
Biotransformation pathways and metabolic derivatives of mitragynine (Kruegel et al., 2019).

The alkaloid composition of Mitragyna speciosa primarily includes mitragynine and 7-hydroxymitragynine (7-OH), which are considered the key contributors to its pharmacological effects (Fig. 1). Mitragynine constitutes 1–2 % of the dry leaf mass and up to two-thirds of the total alkaloid content, while 7-OH is present in much lower concentrations, typically less than 0.05 % of the dry leaf mass. Both compounds act as partial agonists of the human μ-opioid receptor (hMOR), with 7-OH being approximately ten times more potent than mitragynine. These alkaloids are also G protein-biased agonists, reducing β-arrestin-2 pathway activation, which is linked to adverse effects of traditional opioids, such as respiratory depression. Interestingly, recent studies have highlighted the potential anticancer properties of G protein-biased agonists, suggesting that their ability to selectively modulate signaling pathways may have therapeutic relevance in cancer treatment, including breast cancer (Kruegel et al., 2019).

The structures of these compounds were retrieved from the PubChem database in sdf format and subsequently prepared for analysis (Kim et al., 2023). Structural optimization and visualization were performed using MarvinSketch to ensure the accuracy of the molecular geometries (ChemAxon, 2022). Further computational refinement was conducted using Gaussian software, where optimization and frequency calculations were carried out to confirm stable geometries and to evaluate vibrational properties (Schlegel et al., 2016). These optimized structures were then evaluated for drug-likeness using Lipinski's rule of five and additional filters, including Ghose, Veber, and Egan criteria, ensuring favorable physicochemical and pharmacokinetic properties (Karami et al., 2022). Finally, the alkaloids were subjected to molecular docking and molecular dynamics simulations to assess their therapeutic potential as HER2 inhibitors in breast cancer.

### 2.3 Molecular docking protocol validation

To validate the docking protocol, the test compounds (Mitragynine and 7-Hydroxymitragynine) were docked against the active site of the HER2 kinase domain (PDB ID: 3PP0). Key amino acid residues within the active site pocket, including Leu726, Val734, Ala751, Lys753, Thr798, Gly804, Arg849, Leu852, Thr862, and Asp863, were identified as crucial interaction points. These residues were consistently observed to interact with previously reported control ligands such as Lapatinib, Afatinib, and Sapitinib, confirming the reliability of the active site model (Shu et al., 2023; Zubair and Bandyopadhyay, 2023). Using this defined active pocket, docking simulations for Mitragynine and 7-Hydroxymitragynine were conducted. Visual analysis of the docking results showed strong alignment with the scoring function employed, demonstrating the protocol's robustness. The binding energies of the two alkaloids were found to be comparable to the control ligands, supporting their potential as lead compounds for further investigation. These findings validate the docking approach and reinforce the hypothesis of Mitragynine and 7-Hydroxymitragynine as promising candidates for HER2 inhibition.

### 2.4 Multiple ligand docking procedure

The docking simulations were conducted using AutoDock (Forli et al., 2012). To prepare the receptor, water molecules and heteroatoms were removed to eliminate potential interferences. Gasteiger charges were added, and hydrogen bonds were optimized to ensure accurate docking predictions. A grid box was defined around the identified active site, with dimensions of 64 Å × 60 Å × 60 Å and centered at coordinates (16.387 Å, 17.394 Å, 26.218 Å). A variety of ligands were tested against the receptor during this study. The docking process was performed in the AutoDock environment using PyRx software, which enabled efficient multiple ligand docking (Pawar and Rohane, 2021). Key parameters such as root mean square deviation (RMSD), the lowest energy conformations, and hydrogen bond interactions were evaluated to determine the binding efficiency of the docked ligands.

### 2.5 Protein-ligand interaction profiling

The Protein Plus server was utilized to generate a detailed 2D interaction profile of the protein-ligand complex. This tool provided insights into the key molecular interactions, including hydrogen bonds, hydrophobic interactions, and π-π stacking, between the ligands and the active site residues of the receptor. The visualization helped identify critical binding residues that contribute to ligand stability within the active pocket. These interactions were further analyzed to assess their significance in ligand binding affinity and specificity. The comprehensive interaction map generated by Protein Plus served as a foundational step for understanding the binding mechanisms and optimizing the selected ligands for further development (Fährrolfes et al., 2017).

### 2.6 Molecular dynamics simulations

Molecular dynamics (MD) simulations were conducted for the HER2-Mitragynine and HER2-7-Hydroxymitragynine complexes using GROMACS v2024.4 for a simulation period of 100 ns (Van Der Spoel et al., 2005; Makarewicz and Kaźmierkiewicz, 2013). The system was solvated using the Simple Point Charge (SPC) water model within a cubic unit cell, ensuring a minimum distance of 10 Å between the protein surface and the box boundary. The GROMOS96 53a6 force field was employed to construct the topologies for the protein-ligand complexes (Schuler et al., 2001). Prior to running the simulation, energy minimization was performed using the steepest descent algorithm for 5000 steps with a convergence criterion of 1000 kcal/mol/nm to eliminate steric clashes and optimize the system's initial configuration. Equilibration was carried out in two phases: the NVT ensemble (constant number of particles, volume, and temperature) and the NPT ensemble (constant number of particles, pressure, and temperature). During these steps, the Berendsen thermostat maintained a stable temperature of 300 K, while the Parrinello-Rahman barostat regulated the pressure at 1 bar. The LINCS (Linear Constraint Solver) algorithm was applied to constrain all bond lengths during the simulation. Following equilibration, a 50 ns production run was performed under the microcanonical (NVE) ensemble with a time step of 2 fs. Trajectories were saved every 10 ps to ensure detailed analysis of the system's behavior over time. The MD results were analyzed to evaluate the stability and dynamics of the protein-ligand complexes. Metrics such as root-mean-square deviation (RMSD), root-mean-square fluctuation (RMSF), radius of gyration (Rg), and solvent-accessible surface area (SASA) were computed to assess structural stability and flexibility. The 2D plots for these parameters were generated using Grace 5.1.23 software, providing clear visual insights into the intrinsic dynamic properties of the complexes.

### 2.7 MM-PBSA analysis

The Molecular Mechanics Poisson-Boltzmann Surface Area (MM-PBSA) method was utilized to estimate the binding free energy of the HER2-Mitragynine and HER2-7-Hydroxymitragynine complexes (Kumari et al., 2014; Wang et al., 2018). This approach combines molecular mechanics energy, solvation effects, and entropic contributions to provide an estimate of the interaction strength between the ligand and the protein. The analysis was carried out using the g_mmpbsa tool integrated within the GROMACS framework. Snapshots from the last 10 ns of the MD simulations were extracted, ensuring that the system had reached equilibrium. A total of 500 frames were selected to capture the dynamic nature of the protein-ligand interactions. The binding free energy was computed as the difference between the free energy of the protein-ligand complex and the sum of the free energies of the individual protein and ligand components. The free energy was decomposed into van der Waals interactions, electrostatic interactions, polar solvation energy, and non-polar solvation energy to identify the key contributors to binding affinity. The polar solvation energy was derived using the Poisson-Boltzmann model, while the non-polar solvation energy was estimated based on the solvent-accessible surface area (SASA).

## 3 Results

The two alkaloids, Mitragynine and 7-Hydroxymitragynine, along with the native ligand (SYR127063), were evaluated for their interactions with HER2 and their ADMET (Absorption, Distribution, Metabolism, Excretion, and Toxicity) profiles. Molecular docking results showed that 7-Hydroxymitragynine demonstrated a higher binding affinity to HER2 with a binding energy of −8.77 kcal/mol, compared to Mitragynine's −7.56 kcal/mol (Table 1). This difference indicates that 7-Hydroxymitragynine may form stronger and more stable interactions within the receptor's active site. However, the native ligand SYR127063 exhibited the highest binding affinity with a binding energy of −10.57 kcal/mol, suggesting an even more stable interaction. The inhibition constant (Ki) values further supported these findings, with 7-Hydroxymitragynine exhibiting a lower Ki of 375.37 nM, compared to Mitragynine's 2.89 μM, indicating greater potency. Notably, SYR127063 displayed the lowest Ki value of 17.84 nM, reinforcing its superior inhibitory potential. The molecular interactions revealed common key residues, including Leu726, Val734, Ala751, and Thr798, contributing significantly to the binding stability of all three ligands. The strong binding energies and inhibitory potential of SYR127063, along with the promising activity of 7-Hydroxymitragynine, highlight their potential as HER2-targeted therapy candidates.

**Table 1:** Interaction energy between Mitragynine and 7-Hydroxymitragynine against HER2.

List of Compounds	PubChem CID	Binding Energy (kcal/mol)	Inhibition Constant
Native (SYR127063)	11684629	−10.57	17.84 nM (nanomolar)

Mitragynine	3034396	−7.56	2.89 μM (micromolar)
7-Hydroxymitragynine	44301524	−8.77	375.37 nM (nanomolar)

The ADMET analysis in Table 2 revealed that both compounds possess favorable pharmacokinetic properties, making them suitable for oral drug development. Both Mitragynine and 7-Hydroxymitragynine demonstrated high gastrointestinal (GI) absorption, a critical parameter for oral bioavailability. Mitragynine was found to be blood-brain barrier (BBB) permeant, suggesting potential CNS effects, whereas 7-Hydroxymitragynine was not BBB permeant, which could reduce the likelihood of CNS-related side effects. Neither compound was classified as a P-glycoprotein (P-gp) substrate, which minimizes the risk of efflux-related bioavailability issues. However, both compounds were inhibitors of CYP2D6 and CYP3A4 enzymes, indicating a potential for drug-drug interactions that should be monitored during co-administration with other medications. Lipinski's Rule of Five was satisfied for both compounds, with no violations, confirming their drug-like nature. These results highlight their potential for development as orally administered drugs while addressing considerations for metabolic interactions.

**Table 2:** ADMET profiles of the selected ligands Mitragynine and 7-Hydroxymitragynine.

Properties	Parameters	Mitragynine	7-Hydroxymitragynine
Physicochemical	Formula	C_23_H_30_N_2_O_4_	C_23_H_30_N_2_O_5_
	Molecular weight	398.50 g/mol	414.49 g/mol
	Num. heavy atoms	29	30
	Num. arom. heavy atoms	9	6
	Fraction Csp 3	0.52	0.57
	Num. rotatable bonds	6	6
	Num. H-bond acceptors	5	7
	Num. H-bond donors	1	1
	Molar Refractivity	116.89	121.14
	TPSA	63.79 Å^2^	80.59 Å^2^
Lipophilicity	Log Po/w (iLOGP)	3.77	3.87
	Log Po/w (XLOGP3)	3.41	2.27
	Log Po/w (WLOGP)	3.12	1.92
	Log Po/w (MLOGP)	2.02	1.30
	Log Po/w (SILICOS-IT)	3.72	3.10
	Consensus Log Po/w	3.21	2.49
Water Solubility	Log S (ESOL)	−4.29	−3.59
	Solubility	2.03e-02 mg/ml; 5.10e-05 mol/l	1.06e-01 mg/ml; 2.56e-04 mol/l
	Class	Moderately soluble	Soluble
	Log S (Ali)	−4.43	−3.60
	Solubility	1.48e-02 mg/ml; 3.72e-05 mol/l	1.04e-01 mg/ml; 2.52e-04 mol/l
	Class	Moderately soluble	Soluble
	Log S (SILICOS-IT)	−5.00	−4.10
	Solubility	3.95e-03 mg/ml; 9.91e-06 mol/l	3.28e-02 mg/ml; 7.91e-05 mol/l
	Class	Moderately soluble	Moderately soluble
Pharmacokinetics	GI absorption	High	High
	BBB permeant	Yes	No
	P-gp substrate	No	No
	CYP1A2 inhibitor	No	No
	CYP2C19 inhibitor	No	No
	CYP2C9 inhibitor	No	No
	CYP2D6 inhibitor	Yes	Yes
	CYP3A4 inhibitor	Yes	Yes
	Log Kp (skin permeation)	−6.31 cm/s	−7.22 cm/s
Druglikeness	Lipinski	Yes; 0 violation	Yes; 0 violation
	Ghose	Yes	Yes
	Veber	Yes	Yes
	Egan	Yes	Yes
	Muegge	Yes	Yes
	Bioavailability Score	0.55	0.55
Medicinal Chemistry	PAINS	0 alert	0 alert
	Brenk	2 alerts: acyclic_C=C-O, michael_acceptor_1	2 alerts: acyclic_C=C-O, michael_acceptor_1
	Leadlikeness	No; 1 violation: MW > 350	No; 1 violation: MW > 350
	Synthetic accessibility	4.49	5.21

**Fig. 2:**
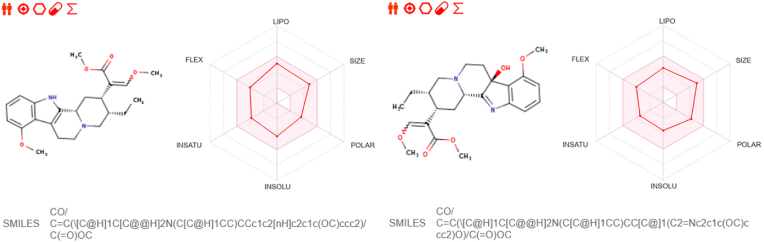
ADMET properties and structural profiles of Mitragynine (Left) and 7-Hydroxymitragynine (Right).

The physicochemical properties of the two alkaloids further validate their potential as drug candidates. Mitragynine and 7-Hydroxymitragynine have molecular weights of 398.50 g/mol and 414.49 g/mol, respectively, which fall within the acceptable range for small molecules. The calculated topological polar surface area (TPSA) values were 63.79 Å^2^ for Mitragynine and 80.59 Å^2^ for 7-Hydroxymitragynine, indicating their suitability for cell membrane permeability and solubility. Solubility classification revealed that Mitragynine was moderately soluble, whereas 7-Hydroxymitragynine was classified as soluble, suggesting the latter's potential for improved bioavailability. Both compounds were free from PAINS (Pan Assay Interference) alerts, ensuring that their interactions are specific to HER2 and less likely to cause off-target effects. However, Brenk alerts were identified for acyclic structures in both compounds, which may require optimization to improve their stability and pharmacological properties. Overall, the physicochemical characteristics of these alkaloids align with drug-likeness criteria, making them strong candidates for further development (Fig. 2).

Drug-likeness assessments using various filters such as Ghose, Veber, Egan, and Muegge rules further confirmed the suitability of these compounds as potential drugs (Table 3). Both compounds passed all these filters, demonstrating their favorable physicochemical and pharmacokinetic profiles. Synthetic accessibility scores of 4.49 for Mitragynine and 5.21 for 7-Hydroxymitragynine indicate moderate feasibility for large-scale synthesis. This is a critical factor when considering the production of drug candidates for preclinical and clinical trials. The combination of favorable drug-likeness properties and moderate synthetic accessibility supports their advancement in the drug discovery pipeline. These findings suggest that Mitragynine and 7-Hydroxymitragynine hold significant promise for further experimental evaluation as HER2-targeted therapeutics.

**Table 3:** Various guidelines for drug-like molecule classification.

Properties	Parameters	Mitragynine	7-Hydroxymitragynine
Lipinski Rule of Five	Molecular mass: <500 Dalton	Yes	Yes
	High lipophilicity: LogP <5		
	Hydrogen bond donors: <5		
	Hydrogen bond acceptors: <10		
	Molar refractivity: 40 to130		
Ghose Rule	clogP: −0.4 to 5.6	Yes	Yes
	MW: 160 to 480		
	Molar refractivity: 40 to 130		
	Total number of atoms: 20 to 70		
Veber Rule	Rotatable bonds: ≤10	Yes	Yes
	Polar surface area: ≤140 Å^2^		
Egan Rule	WlogP: ≤5.88	Yes	Yes
	TPSA: ≤131.6		
Muegge Rule	MW: 200 to 600	Yes	Yes
	XlogP: −2 to 5		
	TPSA: ≤150		
	Number of rings: ≤7		
	Number of carbon: >4		
	Number of heteroatoms: >1		
	Number of rotatable bonds: ≤15		
	Hydrogen bond acceptors: ≤10		
	9. Hydrogen bond donors: ≤5		

Therefore, both Mitragynine and 7-Hydroxymitragynine exhibit strong binding affinities and favorable ADMET profiles, making them viable candidates for HER2-targeted breast cancer therapy. However, 7-Hydroxymitragynine demonstrated superior binding energy, solubility, and inhibitory potency compared to Mitragynine, making it the more promising compound of the two. While both compounds meet key drug-likeness criteria, potential CYP enzyme inhibition and Brenk alerts highlight the need for further optimization during lead development. The combined results from docking, ADMET analysis, and drug-likeness assessments provide a robust foundation for advancing these alkaloids into experimental and clinical research. These findings open new avenues for leveraging natural compounds from Mitragyna speciosa in breast cancer treatment.

### 3.1 Molecular interactions between mitragynine and HER2

The free energy of binding for the Mitragynine-HER2 complex was calculated to be −7.56 kcal/mol, indicating moderate stability within the HER2 binding pocket. Interaction analysis, as detailed in Table 4, revealed key interactions with several amino acid residues, including Thr862, Asp863, Ser783, Thr798, Leu726, Val734, Ala751, Lys753, Leu852, and Arg849. Hydrogen bonding played a significant role in stabilizing the complex, with conventional hydrogen bonds observed at Thr862 (2.66 Å) and Asp863 (3.03 Å). Additionally, carbon-hydrogen bonds were formed with Ser783 (3.71 Å), Thr798 (3.16 Å), and Leu726 (3.63 Å), further reinforcing the interaction network. Hydrophobic interactions also contributed significantly, with residues such as Val734, Ala751, Leu852, and Lys753 engaging in alkyl interactions. Furthermore, Pi-Alkyl interactions were observed with residues Val734 and Arg849, stabilizing the ligand's orientation. These diverse interaction types highlight the moderate yet effective binding of Mitragynine to HER2.

**Table 4:** Comprehensive analysis of Mitragynine-HER2 interaction.

Interaction	Distance	Category	Types
A:THR862:OG1	2.6624	Hydrogen Bond	Conventional Hydrogen Bond
A:ASP863:OD2	3.02763	Hydrogen Bond	Conventional Hydrogen Bond
A:SER783:O	3.71354	Hydrogen Bond	Carbon Hydrogen Bond
A:THR798:OG1	3.1625	Hydrogen Bond	Carbon Hydrogen Bond
A:LEU726:O	3.6287	Hydrogen Bond	Carbon Hydrogen Bond
A:VAL734	4.77501	Hydrophobic	Alkyl
A:VAL734	3.83609	Hydrophobic	Alkyl
A:ALA751	4.85024	Hydrophobic	Alkyl
A:ALA751	3.86119	Hydrophobic	Alkyl
A:LEU852	4.94138	Hydrophobic	Alkyl
A:VAL734	4.84259	Hydrophobic	Alkyl
A:LYS753	4.45784	Hydrophobic	Alkyl
A:VAL734	4.78952	Hydrophobic	Pi-Alkyl
A:ARG849	5.18414	Hydrophobic	Pi-Alkyl

**Fig. 3:**
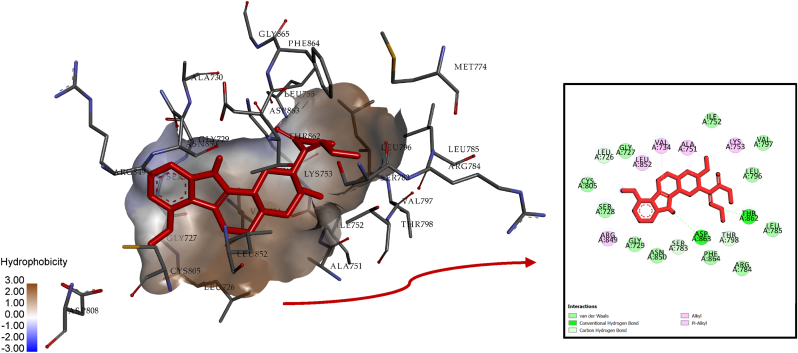
Visualization of Mitragynine-HER2 interaction in 2D and 3D.

Visual analysis of the interaction, as shown in Fig. 3, provides both 2D and 3D representations, illustrating the spatial positioning of Mitragynine within the HER2 binding pocket. Despite its moderate binding energy, Mitragynine forms critical interactions that suggest its potential as a HER2 inhibitor. However, the relatively lower binding energy compared to 7-Hydroxymitragynine indicates room for optimization to enhance its stability and affinity. The combination of hydrogen bonds, hydrophobic contacts, and Pi interactions suggests that Mitragynine engages HER2 effectively, though less robustly than its counterpart. These findings establish Mitragynine as a compound with potential for therapeutic exploration, warranting further refinement and experimental validation to fully realize its potential in targeting HER2.

### 3.2 Molecular interactions between 7-hydroxymitragynine and HER2

For the 7-Hydroxymitragynine-HER2 complex, the binding energy was calculated to be −8.77 kcal/mol, reflecting a stronger binding affinity than Mitragynine. As summarized in Table 5, the key amino acid residues involved in stabilizing the complex included Cys805, Thr862, Asp808, Met801, Val734, Ala751, Leu852, and Thr798. Notably, conventional hydrogen bonds were observed with Cys805 (3.19 Å), Thr862 (1.96 Å), and Asp808 (2.81 Å), indicating strong polar interactions. Carbon-hydrogen bonds were identified with Met801 (3.10 Å) and Asp808 (2.81 Å), further contributing to the ligand's stability within the binding pocket. Hydrophobic interactions were prominent, involving residues such as Val734, Leu852, and Ala751, which formed alkyl interactions. Additionally, Pi-Sigma and Pi-Alkyl interactions were observed with residues such as Thr798, Val734, and Lys753, enhancing the overall binding stability of 7-Hydroxymitragynine.

**Table 5:** Comprehensive analysis of 7-Hydroxymitragynine-HER2 interaction.

Interaction	Distance	Category	Types
A:CYS805:N	3.19147	Hydrogen Bond	Conventional Hydrogen Bond
A:THR862:OG1	1.96333	Hydrogen Bond	Conventional Hydrogen Bond
A:MET801:O	3.1018	Hydrogen Bond	Carbon Hydrogen Bond
A:ASP808:OD1	2.81869	Hydrogen Bond	Carbon Hydrogen Bond
A:THR798:CG2	3.77555	Hydrophobic	Pi-Sigma
A:VAL734	4.33054	Hydrophobic	Alkyl
A:LEU852	4.84603	Hydrophobic	Alkyl
A:VAL734	3.57852	Hydrophobic	Alkyl
A:VAL734	4.70915	Hydrophobic	Pi-Alkyl
A:ALA751	4.6395	Hydrophobic	Pi-Alkyl
A:LYS753	3.92094	Hydrophobic	Pi-Alkyl

**Fig. 4:**
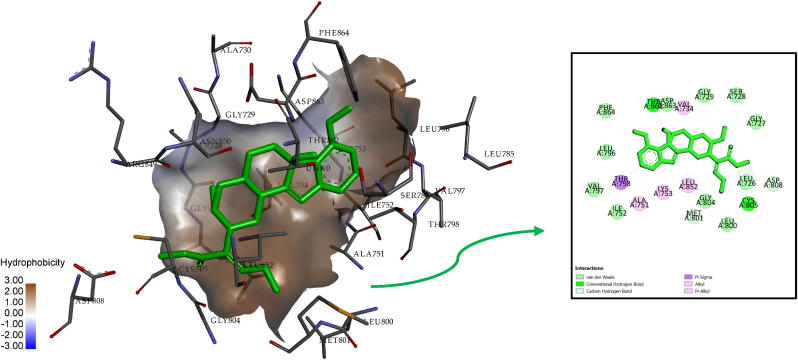
Visualization of 7-Hydroxymitragynine-HER2 interaction in 2D and 3D.

The structural visualization in Fig. 4 provides a clear depiction of the ligand-receptor complex, highlighting the superior binding characteristics of 7-Hydroxymitragynine compared to Mitragynine. The combination of multiple hydrogen bonds, strong hydrophobic interactions, and Pi interactions underscores the ligand's higher affinity and stability. These properties suggest that 7-Hydroxymitragynine is a more promising candidate for HER2-targeted therapy, particularly in breast cancer treatment. The robust interaction profile, coupled with its stronger binding energy, supports its potential as a lead compound. Future studies should focus on further characterizing these interactions through experimental validation and optimizing its structure to enhance specificity and efficacy (Suwendar et al., 2023).

### 3.3 Molecular dynamics simulations outcomes

**Fig. 5:**
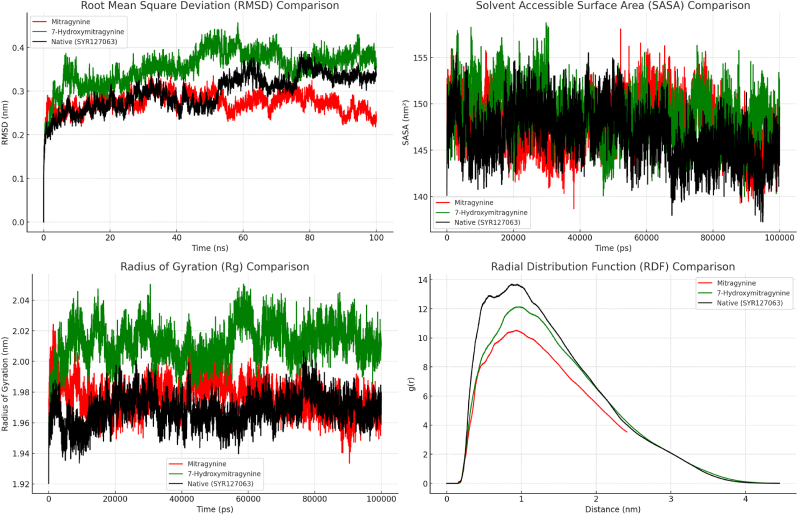
The RMSD, SASA, Rg, and RDF plotted against simulation time for HER2 in complex with Mitragynine (depicted in red), 7-Hydroxymitragynine (depicted in green), and Native (SYR127063) (depicted in black).

The molecular dynamics simulation (MDS) of HER2 complexes with Mitragynine, 7-Hydroxymitragynine, and Native (SYR127063) was analyzed using several key metrics, including Root Mean Square Deviation (RMSD), Solvent Accessible Surface Area (SASA), Radius of Gyration (Rg), and Radial Distribution Function (RDF) (Fig. 5). These analyses provide a comprehensive understanding of the dynamic stability and interaction characteristics of the complexes over the simulation period. The chosen ligands were studied due to their potential inhibitory activity against HER2, a critical target in cancer therapy. MDS is a robust computational method that allows the evaluation of structural fluctuations, binding stability, and dynamic behavior under physiological conditions (Yuniarta et al., 2023). By employing these metrics, differences in ligand binding characteristics and their effects on the structural behavior of HER2 could be thoroughly investigated. The results from these simulations highlight critical aspects of the complexes' stability and interaction patterns. Such data are essential to predict the potential efficacy of these ligands in real-world therapeutic scenarios. The combination of structural and dynamic insights provides a solid foundation for further optimization of these ligands as HER2 inhibitors.

The RMSD values were calculated to evaluate the overall structural stability of HER2 complexes with Mitragynine, 7-Hydroxymitragynine, and Native (SYR127063). RMSD measures the deviation of the complex's atomic positions from the reference structure over time, providing insights into conformational stability. The RMSD plots indicate that all three complexes maintained stability throughout the simulation period, with minor fluctuations suggesting transient structural adjustments. The Mitragynine complex displayed RMSD values that remained relatively stable over time, indicating consistent conformational behavior. The 7-Hydroxymitragynine complex exhibited slightly higher fluctuations, particularly at the beginning of the simulation, likely due to initial adaptation to the HER2 binding site. The Native (SYR127063) complex maintained the most stable RMSD trajectory, with minimal deviations observed. These findings suggest strong interactions between HER2 and the ligands, an essential criterion for potential drug candidates.

The SASA values were analyzed to evaluate the solvent exposure of the HER2 complexes. The SASA plot shows that all three complexes exhibited dynamic fluctuations throughout the simulation. The Mitragynine and 7-Hydroxymitragynine complexes displayed slightly higher SASA variations compared to the Native complex, indicating ligand-induced changes in solvent exposure. These fluctuations suggest dynamic rearrangements in the HER2-ligand binding interface, which could impact binding affinity and stability. The Radius of Gyration (Rg) was used to assess the compactness of the HER2 complexes. The Rg values in All three complexes maintained relatively stable compactness, with minor fluctuations observed. The 7-Hydroxymitragynine complex exhibited the highest Rg values, indicating a slightly expanded conformation compared to the Mitragynine and Native complexes. This suggests potential differences in ligand-induced structural flexibility within the HER2 binding pocket.

The Radial Distribution Function (RDF) was employed to examine the spatial distribution of ligand interactions within the HER2 binding site. The RDF plots reveal distinct interaction patterns among the three complexes. The Native complex exhibited the highest peak, indicating stronger local density interactions compared to Mitragynine and 7-Hydroxymitragynine. The Mitragynine and 7-Hydroxymitragynine complexes displayed lower RDF peaks, suggesting slightly reduced interaction density, which could influence binding stability. In addition, the molecular dynamics simulations provide valuable insights into the stability, flexibility, and interaction characteristics of HER2 complexes with Mitragynine and 7-Hydroxymitragynine. The findings suggest that while both ligands exhibit stable binding, their structural effects on HER2 differ slightly, which could impact their potential efficacy as inhibitors. These results contribute to a deeper understanding of ligand-protein interactions and aid in the rational design of novel HER2-targeting therapeutics.

**Fig. 6:**
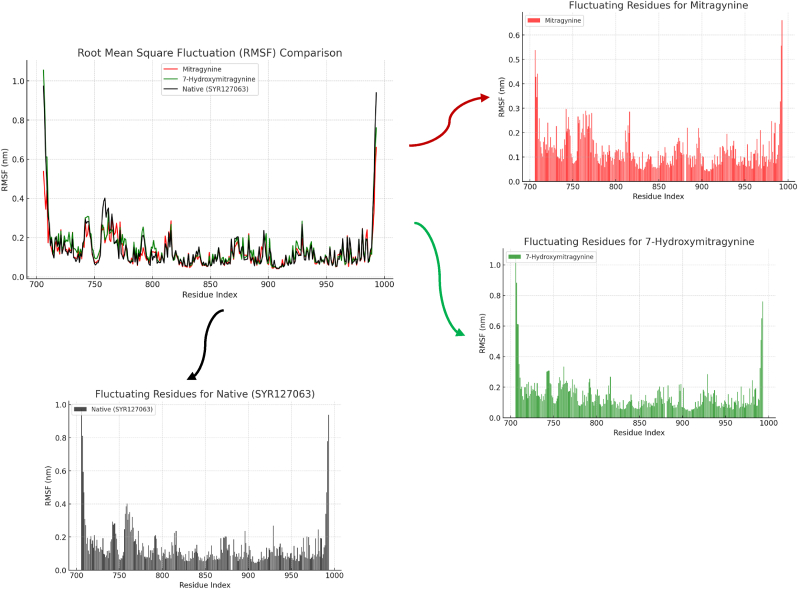
The RMSF values are illustrated over the simulation period for HER2 interacting with Mitragynine (represented in red), 7-Hydroxymitragynine (represented in green), and Native (SYR127063) (represented in black).

The RMSF analysis further elucidated the flexibility of HER2 residues during interactions with the ligands (Fig. 6). This metric measures the average displacement of individual residues from their mean positions, highlighting regions with higher or lower flexibility. The RMSF plots reveal that certain regions of HER2 displayed elevated fluctuations, indicative of localized flexibility. These flexible regions may correspond to loops or non-structured areas that dynamically adapt to ligand binding. Conversely, other regions showed lower RMSF values, representing stable interactions and rigid structural domains. The ligand-binding domains exhibited relatively low flexibility, underscoring their role in stabilizing the complexes. The individual RMSF plots for Mitragynine, 7-Hydroxymitragynine, and Native (SYR127063) further illustrate differences in flexibility profiles. The 7-Hydroxymitragynine complex exhibited slightly higher fluctuations compared to the other two, particularly in certain loop regions, suggesting greater adaptability within the HER2 binding site. Meanwhile, the Native (SYR127063) complex displayed the lowest overall RMSF values, indicative of a more rigid structure. These findings support the idea that both ligands interact dynamically with HER2, though with distinct flexibility profiles, which could influence their binding stability and overall inhibitory potential.

**Fig. 7:**
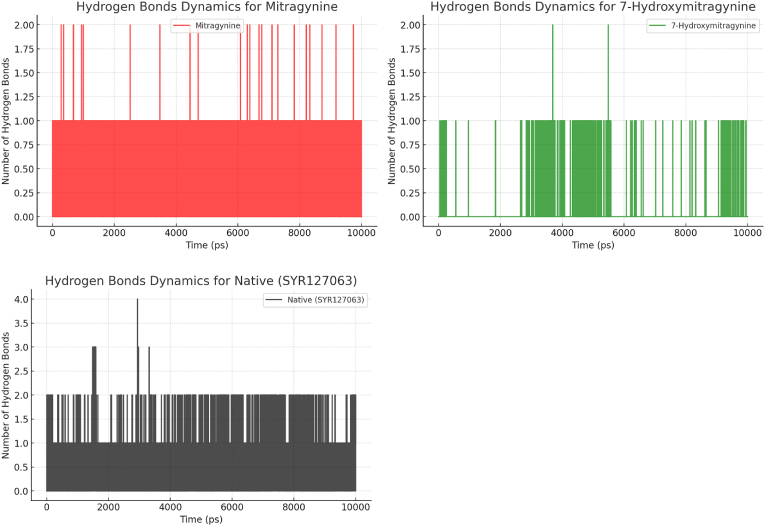
The hydrogen bond dynamics are depicted throughout the simulation timeframe for HER2 in association with Mitragynine (indicated in red), 7-Hydroxymitragynine (indicated in green), and Native (SYR127063) (indicated in black).

Hydrogen bond dynamics play a crucial role in determining the stability and interaction strength of protein-ligand complexes. Analysis of hydrogen bond occupancy in Fig. 7 revealed significant differences between the three complexes. Mitragynine exhibited the highest total hydrogen bond occupancy (39.19 %), with a dominant interaction at ASP863 (38.28 %), and minor contributions from LYS753 (0.54 %) and THR798 (0.37 %). This high occupancy suggests that Mitragynine forms robust and stable hydrogen bonds with HER2. In contrast, 7-Hydroxymitragynine demonstrated a total hydrogen bond occupancy of 4.32 %, primarily due to ASP863 (3.46 %), with minor contributions from THR862 (0.28 %) and LYS753 (0.10 %). The lower hydrogen bond occupancy of 7-Hydroxymitragynine suggests weaker and less stable binding compared to Mitragynine. Native (SYR127063) exhibited a broader distribution of hydrogen bond interactions, with the highest occupancy at ASP863 (19.60 %), followed by MET801 (14.67 %) and ARG849 (7.03 %), contributing to a total occupancy of 49.66 % (Table 6). These results indicate that Native (SYR127063) maintains the strongest hydrogen bonding profile among the three complexes.

**Table 6:** The occupancy patterns of hydrogen bonds during the simulation period for HER2 in complex with Mitragynine, 7-Hydroxymitragynine, and Native (SYR127063).

List of Compounds	Donor	Acceptor	Occupancy	Total
Native (SYR127063)	MET801-Main	Native (SYR127063)-Side	14.67 %	49.66 %
	Native (SYR127063)-Side	ARG849-Main	7.03 %	
	Native (SYR127063)-Side	ASN850-Side	0.19 %	
	Native (SYR127063)-Side	ASP863-Side	19.60 %	
	Native (SYR127063)-Side	SER728-Main	3.55 %	
	Native (SYR127063)-Side	LEU726-Main	0.27 %	
	SER728-Main	Native (SYR127063)-Side	0.26 %	
	LYS753-Side	Native (SYR127063)-Side	0.05 %	
	Native (SYR127063)-Side	GLY727-Main	0.03 %	
	CYS805-Main	Native (SYR127063)-Side	0.71 %	
	Native (SYR127063)-Side	ASP808-Side	2.88 %	
	Native (SYR127063)-Side	SER728-Side	0.01 %	
	GLY727-Main	Native (SYR127063)-Side	0.26 %	
	Native (SYR127063)-Side	VAL851-Main	0.15 %	
Mitragynine	Mitragynine-Side	ASP863-Side	38.28 %	
	LYS753-Side	Mitragynine-Side	0.54 %	39.19 %
	THR798-Side	Mitragynine-Side	0.37 %	
7-Hydroxymitragynine	CYS805-Main	7-Hydroxymitragynine-Side	0.12 %	
	THR798-Side	7-Hydroxymitragynine-Side	0.11 %	
	7-Hydroxymitragynine-Side	ASP863-Side	0.23 %	
	LYS753-Side	7-Hydroxymitragynine-Side	0.10 %	4.32 %
	7-Hydroxymitragynine-Side	ASP863-Main	3.46 %	
	7-Hydroxymitragynine-Side	THR862-Side	0.28 %	
	LYS736-Side	7-Hydroxymitragynine-Side	0.01 %	
	SER783-Side	7-Hydroxymitragynine-Side	0.01 %	

Complementary analyses using RMSD, RMSF, SASA, Rg, RDF, and hydrogen bond occupancy provided additional insights into the complexes' stability and structural behavior. RMSD and RMSF analyses highlighted differences in overall conformational stability and local flexibility, demonstrating that Mitragynine and Native (SYR127063) complexes maintained lower fluctuations compared to 7-Hydroxymitragynine. SASA analysis indicated differences in solvent exposure, where Mitragynine formed a more compact and less solvent-accessible complex compared to 7-Hydroxymitragynine. Rg measurements confirmed these findings, with Mitragynine maintaining a consistently lower Rg value, indicative of a more tightly packed complex. RDF analysis revealed the spatial distribution of ligand interactions, further supporting the superior binding characteristics of Mitragynine. The hydrogen bond analysis reinforced these findings, showing that Mitragynine exhibited stronger and more stable hydrogen bonding interactions than 7-Hydroxymitragynine. Collectively, these metrics demonstrate that Mitragynine exhibits stronger and more stable interactions with HER2 than 7-Hydroxymitragynine. This enhanced stability is critical for effective inhibition, as it ensures sustained binding under physiological conditions. The Native (SYR127063) complex exhibited the highest overall interaction stability, highlighting its role as a control in understanding ligand-induced structural changes. These findings emphasize the potential of Mitragynine as a lead compound for targeting HER2, paving the way for further optimization and development. The integration of multiple analytical approaches strengthens the validity of these conclusions and underscores the robustness of the simulation results.

### 3.4 MM-PBSA analysis

**Fig. 8:**
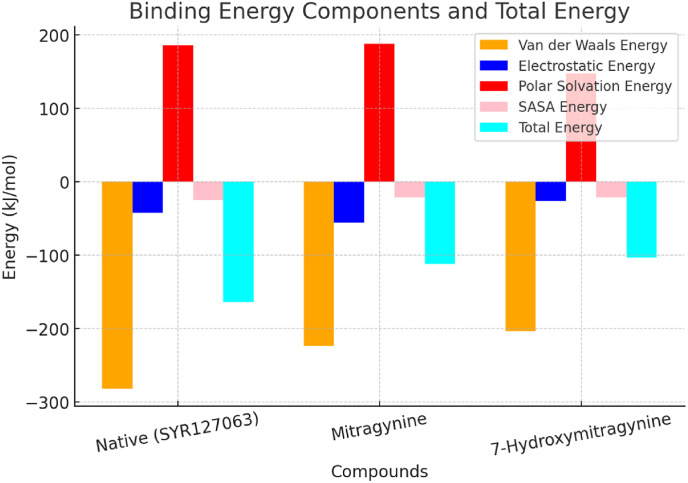
Energy contributions of HER2 complexes with Mitragynine and 7-Hydroxymitragynine.

The binding energy analysis of HER2 complexes with Mitragynine, 7-Hydroxymitragynine, and Native (SYR127063), as shown in Table 7 and Fig. 8, reveals distinct differences in their interaction dynamics. The van der Waals energy for Mitragynine (−223.326 ± 13.733 kJ/mol) is lower than that of 7-Hydroxymitragynine (−203.197 ± 13.494 kJ/mol), indicating stronger hydrophobic interactions between Mitragynine and HER2. Similarly, the electrostatic energy for Mitragynine (−55.534 ± 9.962 kJ/mol) is more negative compared to 7-Hydroxymitragynine (−26.201 ± 11.687 kJ/mol), suggesting that Mitragynine forms more stable charge-based interactions. These contributions are critical in stabilizing the binding of Mitragynine within the HER2 pocket. While both ligands demonstrate negative values for these components, Mitragynine consistently shows stronger interactions, highlighting its superior binding capability. The favorable van der Waals and electrostatic contributions reinforce the idea that Mitragynine has a stronger affinity for HER2 compared to 7-Hydroxymitragynine. These components are essential for the stability of the complex, particularly in hydrophobic and polar regions of the receptor-ligand interface. The more negative values for Mitragynine suggest a better fit within the HER2 active site, which enhances binding stability.

**Table 7:** Binding energy components and total energy values of HER2 complexes with Mitragynine, 7-Hydroxymitragynine, and Native (SYR127063).

List of Compounds	Van der Waals Energy	Electrostatic Energy	Polar Solvation Energy	SASA Energy	Total Energy
Native (SYR127063)	−281.774±12.575	−42.517±16.483	185.562±18.865	−24.719±0.855	−163.448±17.288
Mitragynine	−223.326±13.733 kJ/mol	−55.534±9.962 kJ/mol	187.906±18.902 kJ/mol	−21.375±0.929 kJ/mol	−112.330±22.412 kJ/mol
7-Hydroxymitragynine	−203.197±13.494 kJ/mol	−26.201±11.687 kJ/mol	147.570±20.493 kJ/mol	−21.729±0.829 kJ/mol	−103.556±15.607 kJ/mol

The polar solvation energy and solvent-accessible surface area (SASA) values further highlight the differences between the three complexes. The polar solvation energy, which represents the cost of desolvation, is highest for Native (SYR127063) (185.562 ± 18.865 kJ/mol), followed by Mitragynine (187.906 ± 18.902 kJ/mol) and 7-Hydroxymitragynine (147.570 ± 20.493 kJ/mol). This indicates that Native (SYR127063) undergoes the most significant desolvation penalty, likely due to strong solvent interactions. The SASA energy, which measures the solvent exposure of the ligand and receptor interface, varies across the three complexes. Native (SYR127063) exhibits the highest SASA energy (−24.719 ± 0.855 kJ/mol), whereas Mitragynine (−21.375 ± 0.929 kJ/mol) and 7-Hydroxymitragynine (−21.729 ± 0.829 kJ/mol) display similar values. This suggests that the latter two ligands occupy comparable binding sites and exhibit similar solvent exposure. However, the combination of higher polar solvation energy and solvent exposure in Native (SYR127063) suggests a more dynamic and solvent-exposed interaction. These energetic differences reinforce the conclusion that Mitragynine forms a more stable complex due to its ability to balance strong binding interactions with desolvation costs.

The total binding energy values confirm the superior binding affinity of Mitragynine compared to 7-Hydroxymitragynine. Mitragynine exhibits a total energy of −112.330 ± 22.412 kJ/mol, which is more favorable than the −103.556 ± 15.607 kJ/mol observed for 7-Hydroxymitragynine. Native (SYR127063) shows the most negative total energy (−163.448 ± 17.288 kJ/mol), indicating the strongest binding stability among the three complexes. This reflects the cumulative contributions of stronger van der Waals and electrostatic interactions, particularly in Native (SYR127063) and Mitragynine, which outweigh the higher polar solvation energy. The more favorable total energy for Native (SYR127063) underscores its enhanced stability and binding efficiency as a HER2 reference ligand. Additionally, the energy components suggest that Native (SYR127063) interacts extensively through hydrophobic and charge-based forces, reinforcing its stability. In contrast, 7-Hydroxymitragynine shows weaker contributions across all components, leading to a less stable complex. These findings strongly support the potential of Mitragynine as a lead compound for HER2 inhibition. Its ability to form stronger interactions and maintain stability under physiological conditions makes it a promising candidate for further development in therapeutic applications.

## 4 Discussion

Natural compounds, such as those derived from Mitragyna speciosa (kratom), have shown immense potential in drug discovery, particularly for developing novel therapeutic agents. Kratom, a tropical evergreen tree native to Southeast Asia, has a long history of traditional use as a natural remedy (Goh et al., 2021; Leksungnoen et al., 2022). The leaves of kratom are consumed in various forms, including teas, powders, and extracts, offering analgesic and psychoactive effects. Among the numerous alkaloids in kratom, mitragynine is the most abundant and widely studied for its pharmacological activity. Upon metabolism, Mitragynine is converted into 7-Hydroxymitragynine, a more potent compound with distinct pharmacodynamic properties. These alkaloids interact with various receptors, including opioid, adrenergic, and serotonergic receptors, which underpins their therapeutic potential. The unique pharmacological profile of kratom alkaloids, particularly their partial agonism at μ-opioid receptors and antagonism at κ- and δ-opioid receptors, makes them attractive candidates for drug development targeting conditions like pain, opioid withdrawal, and cancer.

Several studies have demonstrated the anticancer potential of alkaloid compounds derived from Mitragyna speciosa. Research findings indicate that speciophylline, corynoxine A, and corynoxine B exhibit the most favorable free binding energy (ΔG) values for the estrogen receptor alpha (ERα). Meanwhile, mitraphylline, mitrafoline, and corynoxine B show strong interactions with the P53 protein. ADMET predictions using pkCSM suggest that these alkaloid compounds have high lipophilicity and good permeability. This characteristic enhances their ability to penetrate intestinal and skin cell membranes. Additionally, speciophylline, mitraphylline, and mitrafoline are predicted to be non-hepatotoxic. Therefore, speciophylline and mitraphylline are promising anticancer candidates by inhibiting the estrogen receptor alpha and MDM2 receptor (Priatna et al., 2022). Another study found that crude and alkaloid extracts from kratom leaves enhance A549 human lung cancer cell sensitivity to low-dose doxorubicin (0.1 ppm). The combination of crude extract and doxorubicin increased A549 cell sensitivity by 1.3–2.4 times, while the alkaloid extract combination resulted in a 2.6- to 3.4-fold increase (Bayu et al., 2024).

Furthermore, mitragynine and 7-hydroxymitragynine, along with their related compounds, hold significant potential in drug discovery. These compounds could serve as alternatives to existing drugs and play a role in disease treatment and prevention. Animal studies suggest that mitragynine pseudoindoxyl, an analog of mitragynine, might overcome the side effects of conventional opioid therapies. These side effects include antinociceptive tolerance, physical dependence, and respiratory depression. The pharmacological properties of mitragynine and its derivatives indicate their broad therapeutic applications. Their interaction with multiple receptors, including opioid, adrenergic, and serotonergic receptors, contributes to their diverse biological activity (Begum et al., 2025). This versatility makes them valuable candidates for further drug development. Future research should focus on optimizing these compounds to enhance their efficacy and safety profiles. With further validation, kratom-derived alkaloids could provide novel therapeutic solutions in various medical fields.

The integration of kratom-derived compounds into cancer therapy offers promising prospects, particularly in targeting proteins like HER2, a receptor implicated in aggressive breast cancer. Mitragynine and 7-Hydroxymitragynine's ability to interact with multiple biological pathways, including those related to adrenergic and serotonergic systems, broadens their therapeutic potential. Unlike conventional chemotherapeutic agents, kratom alkaloids exhibit unique mechanisms of action, such as reduced activation of the β-arrestin-2 pathway, which is associated with the adverse effects of traditional opioids like respiratory depression and constipation (Hossain et al., 2023; Annuar et al., 2024). Additionally, their binding versatility may enable them to modulate critical pathways involved in cancer progression and metastasis. The structural diversity and bioactivity of Mitragynine and 7-Hydroxymitragynine highlight the potential for leveraging kratom's chemical scaffolds in the design of HER2-targeted inhibitors with fewer side effects and broader biological activity.

To address the validity of the experimental control, the reference compound Native (SYR127063) has been previously studied for its potent interaction with HER2. Guo et al. (2018) demonstrated that SYR127063 is a HER2-selective ligand capable of stabilizing HER2 dimerization via specific binding to its extracellular domain, showing robust anticancer activity in HER2-overexpressing cell lines such as SKBR3 and BT-474. Additionally, SYR127063 has been shown to inhibit downstream PI3K/AKT signaling pathways, further validating its therapeutic relevance. Another study by Seetaha et al. (2020) used molecular modeling and dynamic simulation to confirm the strong binding affinity and stable complex formation of SYR127063 with HER2. Therefore, the use of SYR127063 as a reference ligand in this study is justified, and it provides a benchmark for evaluating the binding performance of novel kratom-derived compounds. The observed stronger van der Waals and electrostatic interactions of Mitragynine compared to SYR127063 in our MM-PBSA results further suggest the potential superiority of kratom alkaloids under specific binding environments.

While the pharmacokinetic profiles of kratom alkaloids are still being elucidated, the increasing use of kratom globally underscores the need for more comprehensive studies. Existing research has demonstrated the plasma concentrations, half-lives, and pharmacokinetics of Mitragynine and 7-Hydroxymitragynine, providing a foundation for understanding their absorption, metabolism, and distribution. However, controlled studies are limited, particularly regarding their long-term effects and potential drug interactions. Future research should focus on integrating pharmacokinetics with computational and experimental studies to optimize these compounds as therapeutic agents (Sultana et al., 2023; Vora et al., 2023). The ability of Mitragynine and 7-Hydroxymitragynine to interact dynamically with HER2, combined with their favorable pharmacological profiles, positions them as promising candidates for innovative cancer therapies. With further refinement and validation, kratom-derived compounds could offer a novel, natural approach to addressing HER2-driven cancers, complementing existing treatment paradigms.

## 5 Conclusions

In silico approaches provide an efficient and cost-effective way to evaluate drug candidates prior to experimental validation. This study investigated Mitragynine, 7-Hydroxymitragynine, and Native (SYR127063), natural alkaloids from Mitragyna speciosa, as potential HER2 inhibitors using molecular docking, molecular dynamics simulations, and MM-PBSA analysis. The MM-PBSA results showed that Native (SYR127063) had the most favorable total binding energy (−163.448 ± 17.288 kJ/mol), followed by Mitragynine (−112.33 ± 22.41 kJ/mol) and 7-Hydroxymitragynine (−103.56 ± 15.61 kJ/mol), indicating that Native (SYR127063) exhibited the strongest interaction stability. Mitragynine also demonstrated higher hydrogen bond occupancy (39.19 %) than 7-Hydroxymitragynine (4.32 %), supporting its superior binding stability over the latter. These findings, combined with strong van der Waals and electrostatic contributions, reinforce Mitragynine's potential as a promising HER2-targeted therapy. Future experimental validation and structural optimization studies are recommended to further explore and refine these compounds for potential clinical applications.

## CRediT authorship contribution statement

**Nabila Hadiah Akbar:** Conceptualization, Supervision, Study design, Formal analysis, and, Writing – review & editing. **Farendina Suarantika:** Data curation, Validation, and, Writing – review & editing. **Taufik Muhammad Fakih:** Formal analysis, Data curation, and critical manuscript revision. **Ariranur Haniffadli:** Data curation, Methodology, and refinement of research outcomes. **Khoirunnisa Muslimawati:** Data curation, Validation, and contribution to scientific discussions. **Aditya Maulana Perdana Putra:** Data curation, Methodology, and final manuscript refinement. All authors have read and approved the final version of the manuscript and declare that there are no conflicts of interest related to this research.

## Declaration of competing interest

The authors declare that they have no known competing financial interests or personal relationships that could have appeared to influence the work reported in this paper.

## Data Availability

The authors do not have permission to share data.
